# Supplementary material for: Demonstration of an AI-driven workflow for autonomous high-resolution scanning microscopy
Source: Nat Commun. 2023 Sep 7;14:5501. doi: 10.1038/s41467-023-40339-1 (PMC10485018; doi:10.1038/s41467-023-40339-1)
Supplement: Supplementary file 1 — Supplementary Information [file 41467_2023_40339_MOESM1_ESM.pdf]

Supplementary Information:

Demonstration of an AI-driven workflow for autonomous high-resolution scanning microscopy

Saugat Kandel[1,*], Tao Zhou[2], Anakha V Babu[3], Zichao Di[4], Xinxin Li[2,5], Xuedan Ma[2,5], Martin Holt[2], Antonino Miceli[1], Charudatta Phatak[6], and Mathew J. Cherukara[1,*]

[1] Advanced Photon Source, Argonne National Laboratory, Lemont, IL 60439, USA.
[2] Nanoscience and Technology Division, Argonne National Laboratory, Lemont, IL 60439, USA.
[3] KLA Corporation, Ann Arbor, MI 48105, USA.
[4] Mathematics and Computer Science, Argonne National Laboratory, Lemont, IL 60439, USA.
[5]Consortium for Advanced Science and Engineering, University of Chicago, Chicago, Illinois 60637, USA.
[6]Materials Science Division, Argonne National Laboratory, Lemont, IL 60439, USA.
[*]Corresponding authors: Saugat Kandel at skandel@anl.gov; Mathew J. Cherukara at mcherukara@anl.gov

July 7, 2023

S.1 Effect of batch size on FAST results

Figure S.1 shows a comparison of the FAST reconstructions for scan batch size of 1 (FAST-1) and 50 (FAST-50) as a function of the scan coverage for the numerical simulation described in Section 2.2. We observe that FAST-1 initially performs better, with lower NRMSE and higher SSIM, than FAST-50, but this advantage erodes quickly. We ended the FAST-1 experiment at ≈8.2 % sampling.due due to simulation time limitations.

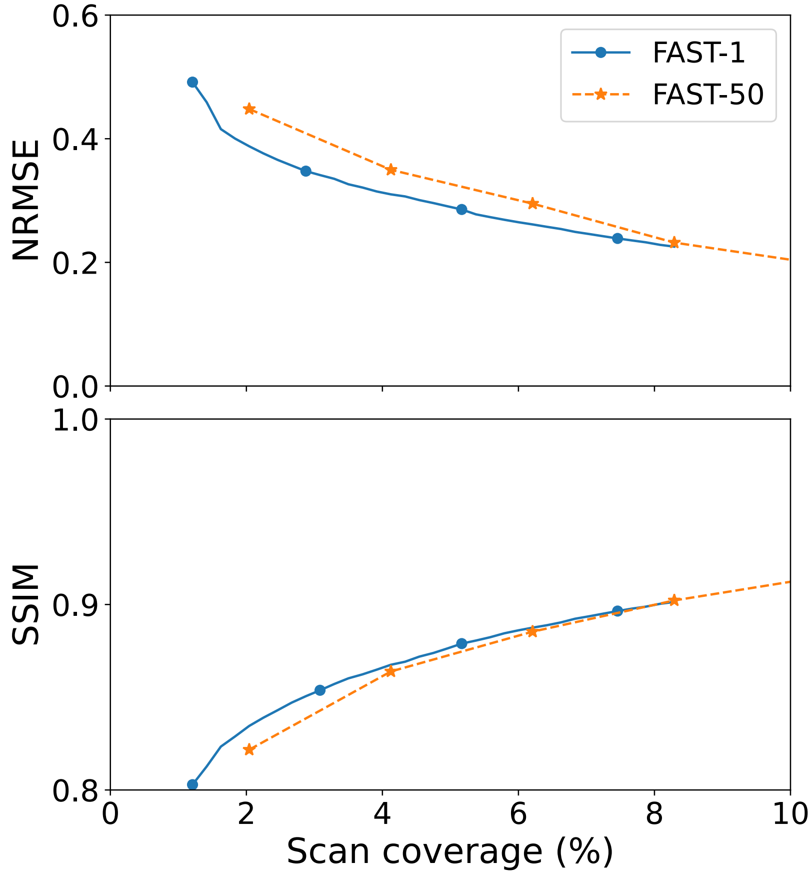

Figure S.1: Comparison of FAST results for batch size of 1 and 50: the normalized root mean squared error (NRMSE) from the ground truth (top), and the structural similarity (SSIM) to the ground truth (bottom). Source data are provided as a Source Data file.

S.2 Choice of reference image for the SLADS-NET training

Before applying the FAST algorithm, we have to train the underlying SLADS-Net neural network on a reference image to generate a trained NN model. Following the reviewers' suggestions, we have applied the training procedure (Section 4.2) individually on 11 miscellaneous images from the USC-SIPI image database, the Scikit-image project, and the MIT Libraries Digital Collection[1, 2, 3]. The images used are: i) female (USC-SIPI 4.1.01), ii) house (USC-SIPI 4.1.05), iii) jellybeans (USC-SIPI 4.1.07), iv) mandrill (USC-SIPI 4.2.03), v) lake (USC-SIPI 4.2.06), vi) peppers (USC-SIPI 4.2.07), vii) male (USC-SIPI 5.3.01), viii) clock (USC-SIPI 5.1.12), ix) plane (USC-SIPI 5.1.11), x) camera (Scikit-image), and xi) cameraman (MIT Libraries). Each of these images was converted to grayscale and resized to 128×128 pixels for the training. In addition to these individual images, we also used the entire collection to train a "combined" SLADS-Net model, thus generating a total of 12 trained NN models. We used these models (which are available in [4]) for the numerical simulation described in Section 2.2, which we shall refer to as the "Flakes" simulation, and an additional numerical simulation on a 128×128 pixel Shepp-Logan phantom. We note that all the Shepp-Logan images used in this work contain added white noise with standard deviation of 0.5 %. Othewrise, all of these tests follow the procedure described in Section 2.2.

In both these tests, the models were all similarly effective at the initial identification of the locations of the features (up to about 15 % of the scan), then diverged in how they cover the sparse and low-contrast regions in the image. We want to highlight that all these models showed excellent performance at finding and scanning the high-contrast regions of the object. We can see this in Figure S.2 that shows the progression in the SSIMs with scan coverage for the two numerical experiments, where the scans show similar progression up to SSIM of $\approx$90 %. Figure S.3 shows the scan points and reconstructions obtained after a 15 % scan for the Shepp-Logan Phantom.

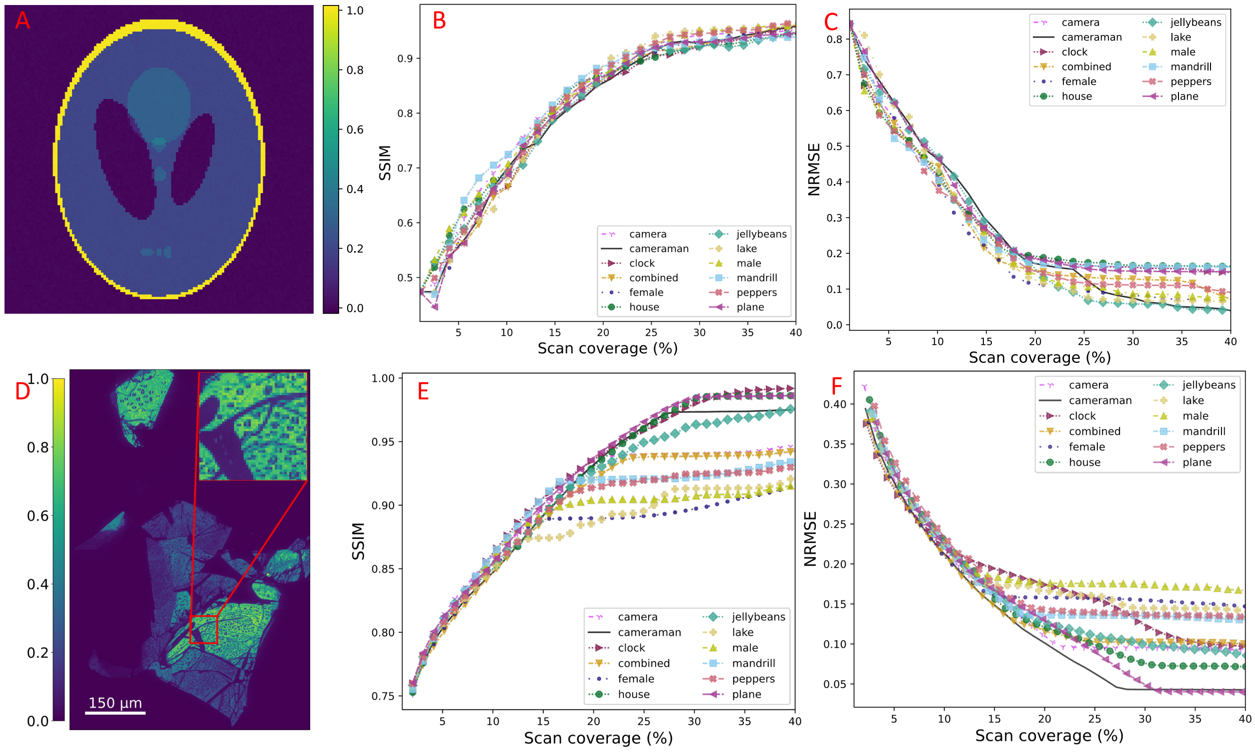

Figure S.2: Comparison of FAST results with different training models: (A) shows the ground truth for the Shepp-Logan image, (B) shows the progression in the SSIM, and (C) the NRMSE. (D, E, F) are the corresponding figures for the Flakes simulation. Source data are provided as a Source Data file.

We can see that all the training models resolve the outer boundaries of the phantom, and then start diverging in

their measurements of the lower contrast features. We see similar results in Figure S.4 for the Flakes case, that the models differ in how they scan the sparse and low-contrast regions of the image.

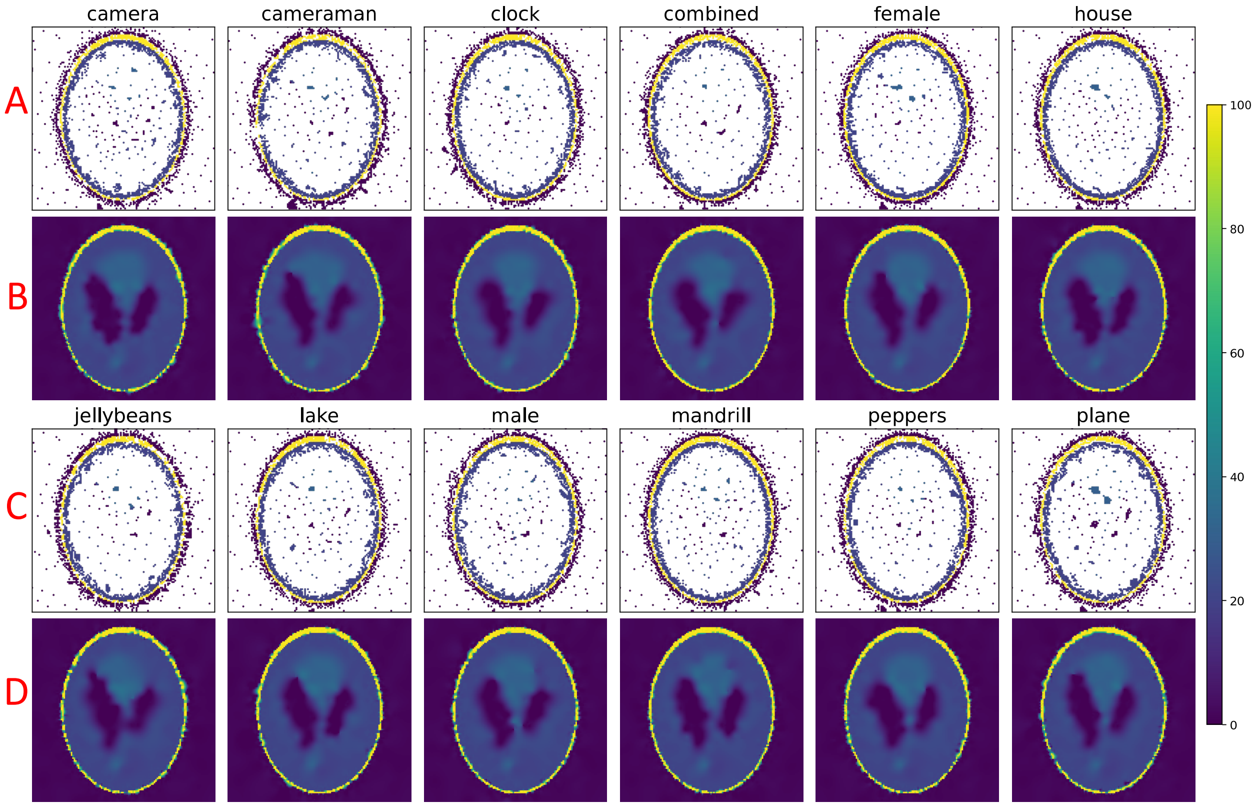

Figure S.3: Measured points and reconstructions for the Shepp-Logan phantom at 15 % scan coverage for FAST scans with the various training models. Individual columns in rows (A,C) show the scan points for the indicated training models and (B,D) show the reconstructions for the measured points in the preceding row.

The fact that these images with a wide variety of structural features can all lead to excellent sparsity performance suggests that the network has learned something intrinsic to image structure and statistics . To analyze this, we looked at the training image intensities, the cumulative distribution function, and the power spectrum of the images (Figure S.5). For the power spectrum calculations, which use the Discrete Fourier Transform of each image, k is the spatial frequency, and $P(k)$ is the power falling within each frequency bin . We see that the test images have varying image statistics, such that we cannot point to one particular feature as the cause for the performance of the FAST scan. Identifying the intrinsic image features that drive the FAST performance is an interesting research question that we plan to explore further in the future.

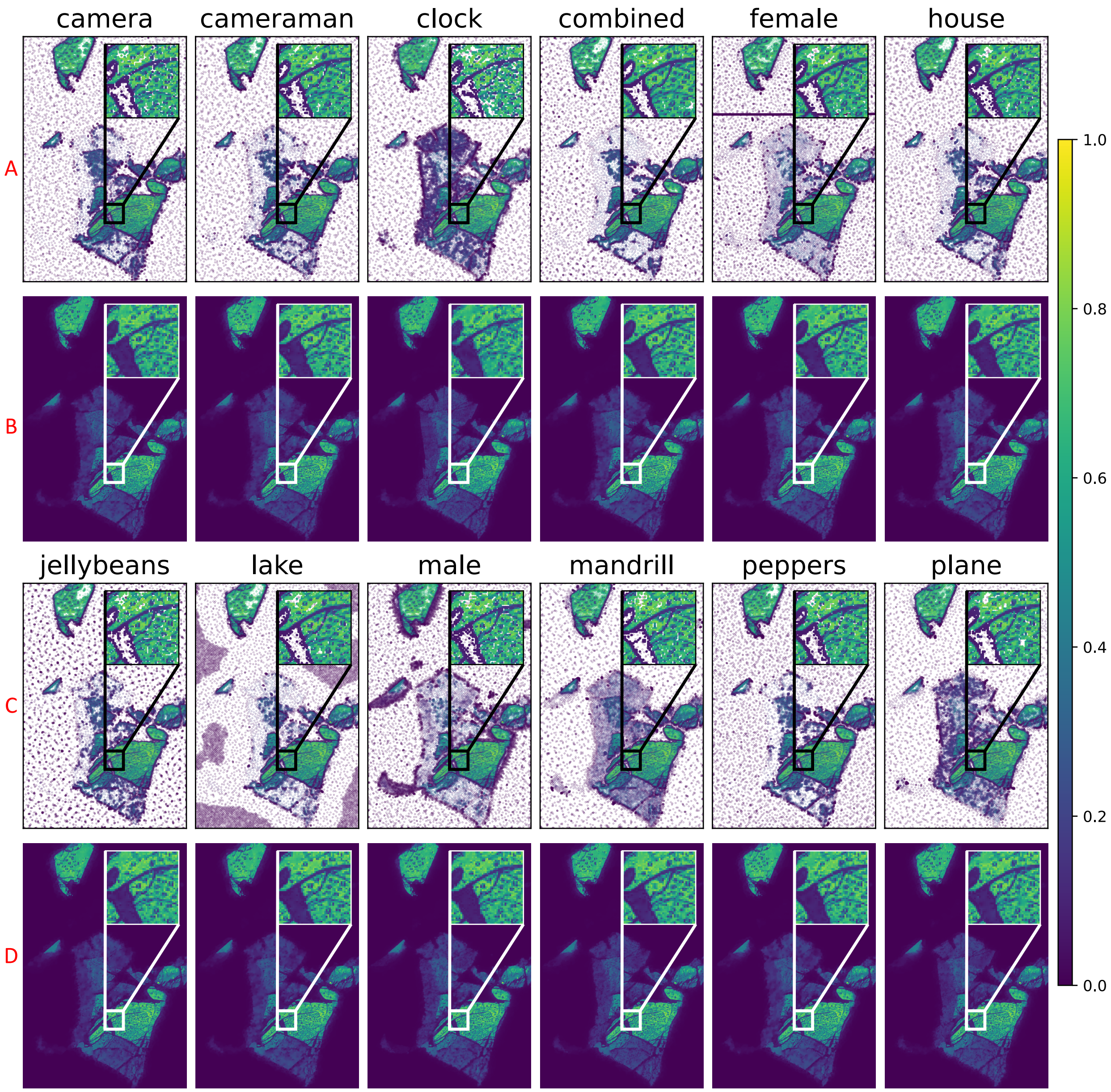

Figure S.4: Measured points and reconstructions for Flakes at 15 % scan coverage for FAST scans with the various training models. Individual columns in rows (A,C) show the scan points for the indicated training models and (B,D) show the reconstructions for the measured points in the preceding row.

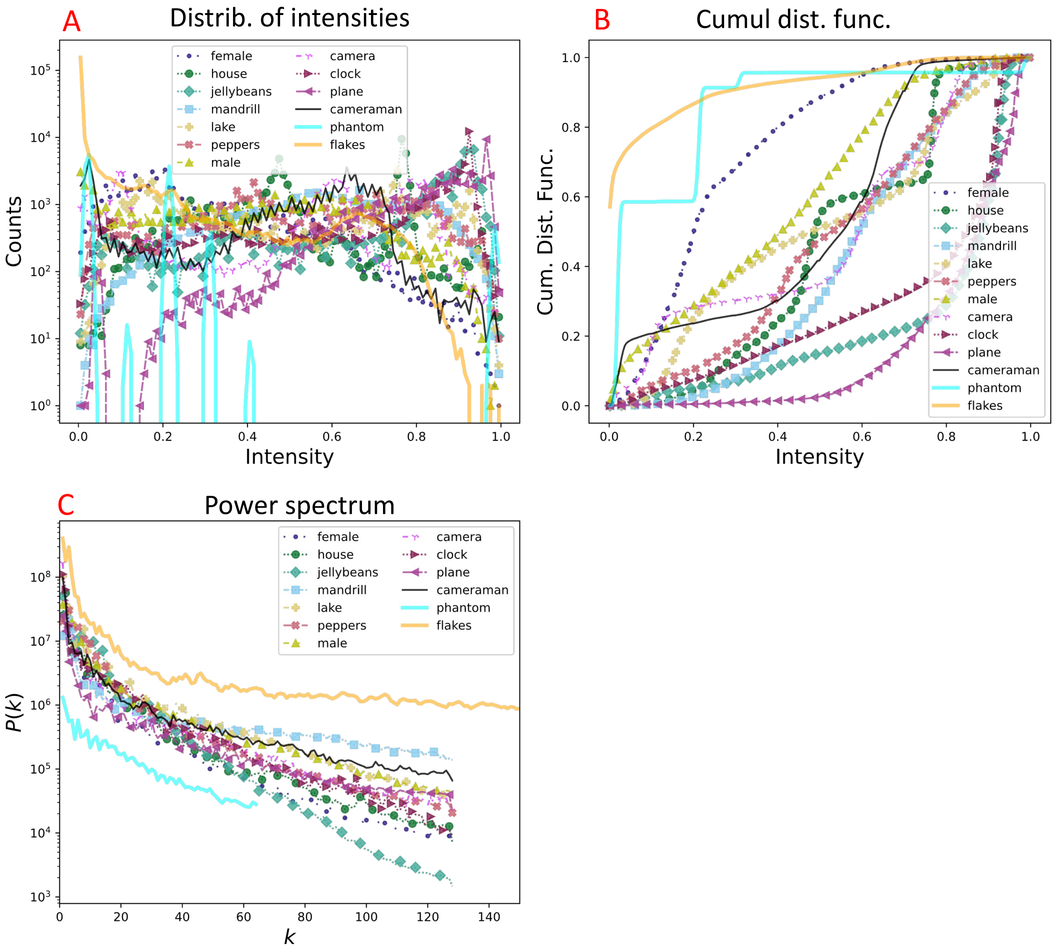

Figure S.5: Image statistics for the various training images and the test images: (A) shows the distribution of the image intensities, (B) the cumulative distribution function for the intensities, and (C) the power spectrum. Source data are provided as a Source Data file.

S.3 Effect of feature sizes, densities, and heterogeneity on FAST performance

To examine how features sizes, densities, and heterogeneity affect FAST performance, we performed two sets of numerical experiments where we: i) change the size of an isolated feature within a 128×128 pixels image, and ii) change the size and number density of the features but ensure that the area covered by the features is largely unchanged. All the experiments use the procedure described in Section 2.2.

S.3.1 Effect of feature sizes for isolated features

We performed five numerical experiments with Shepp-Logan phantoms of size 128×128, 64×64, 32×32, 16×16, and 8×8 pixels, all placed within at the center of 128×128 image. These experiments explore settings with sparse features and varying feature-to-pixel sizes. The full test images, the actual features (cropped), and the FAST results are shown in Figure S.6.

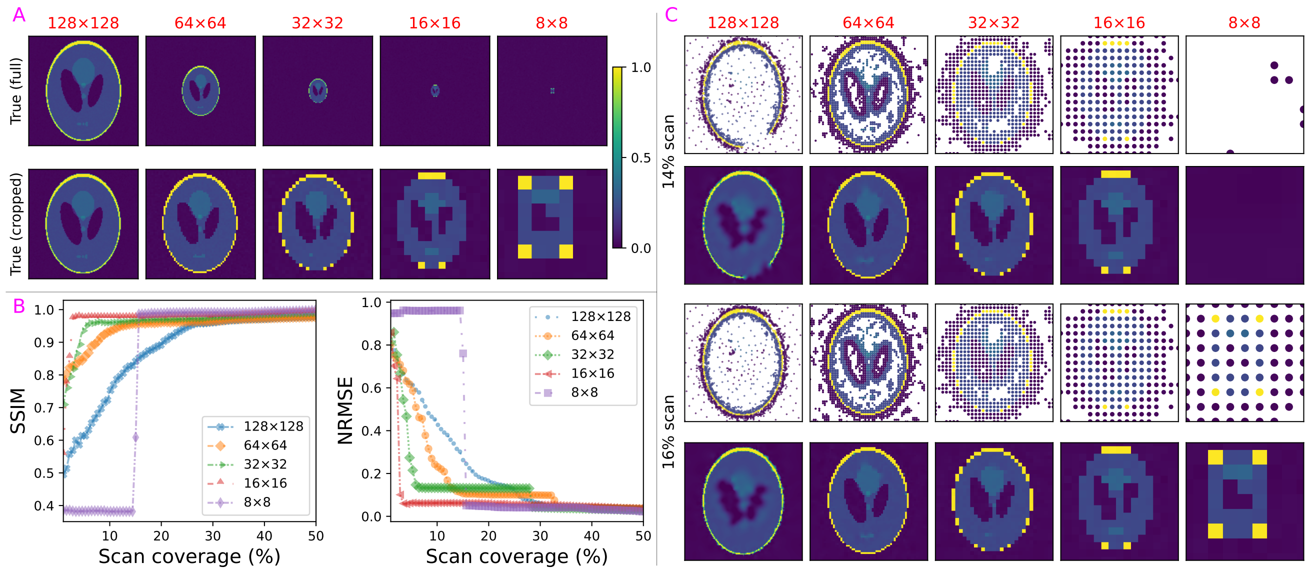

Figure S.6: FAST results for numerical simulations with varying feature sizes. (A) shows the full ground truth images (top row) and only the cropped neighborhood of the features (bottom row) for the different experiments. (B) shows the progression in the SSIM and NRMSE of the indicated cropped regions. (C) shows the scan points and reconstructions of the cropped regions for 14 % scan coverage (top two rows) and 16 % scan coverage (bottom two rows). Source data are provided as a Source Data file.

In Figure S.6, we observe that the efficiency gain through the FAST method increases as the sparsity increases. However, if the isolated feature has a size less than the average spacing between the initial scanpoints, then the feature can be difficult to find. For this experiment, where we have a 128×128 pixels image with a 1 % initial scan, the average spacing between the initial scanpoints is $\approx 12.8 \times 12.8$ pixels. Consequently, the 16×16 phantom is sampled by the initial scan, but the timepoint at which the FAST method samples the 8×8 phantom is random (15 % in this case). Once it identifies the 8×8 phantom, however, FAST immediately prioritizes and accurately reconstructs the phantom.

In fact, if we have advance knowledge of the expected feature size, then we can just calculate the initial scan pattern that ensures that almost every image patch of this size is sampled during the initial scan. Noting that the 8×8 phantom has an actual feature size of 7×5 pixels, we need an initial 6 % scan coverage to ensure that >99 % of image patches of this pixels are sampled during the initial scan. Once we apply this consideration to the initial scan, the FAST method almost always retains an advantage over random, quasi-random, or raster scan grids for scans of isolated features.

The same calculation also informs us that for a 128×128 image, a 1 % quasi-random scan is sufficient to sample 99 % of image patches that are 13×13 pixels in size. The FAST code includes a function to determine the required initial scan coverage based on the expected feature size.

S.3.2 Effect of feature density

We performed numerical experiments with 2×2, 4×4, 8×8 tilings of correspondingly scaled Shepp-Logan phantom, all placed inside a 128×128 image. These experiments explore the case where the area covered by the features remains similar, but the number of the features is different. A consequence of the change in the number of features is that the overall contour, or the perimeter, of the features also changes significantly between the experiments. We present the ground truth images and FAST results in Figure S.7, where the 1×1 case shown in the SSIM and NRMSE figures are from the experiment in Section S.3.1.

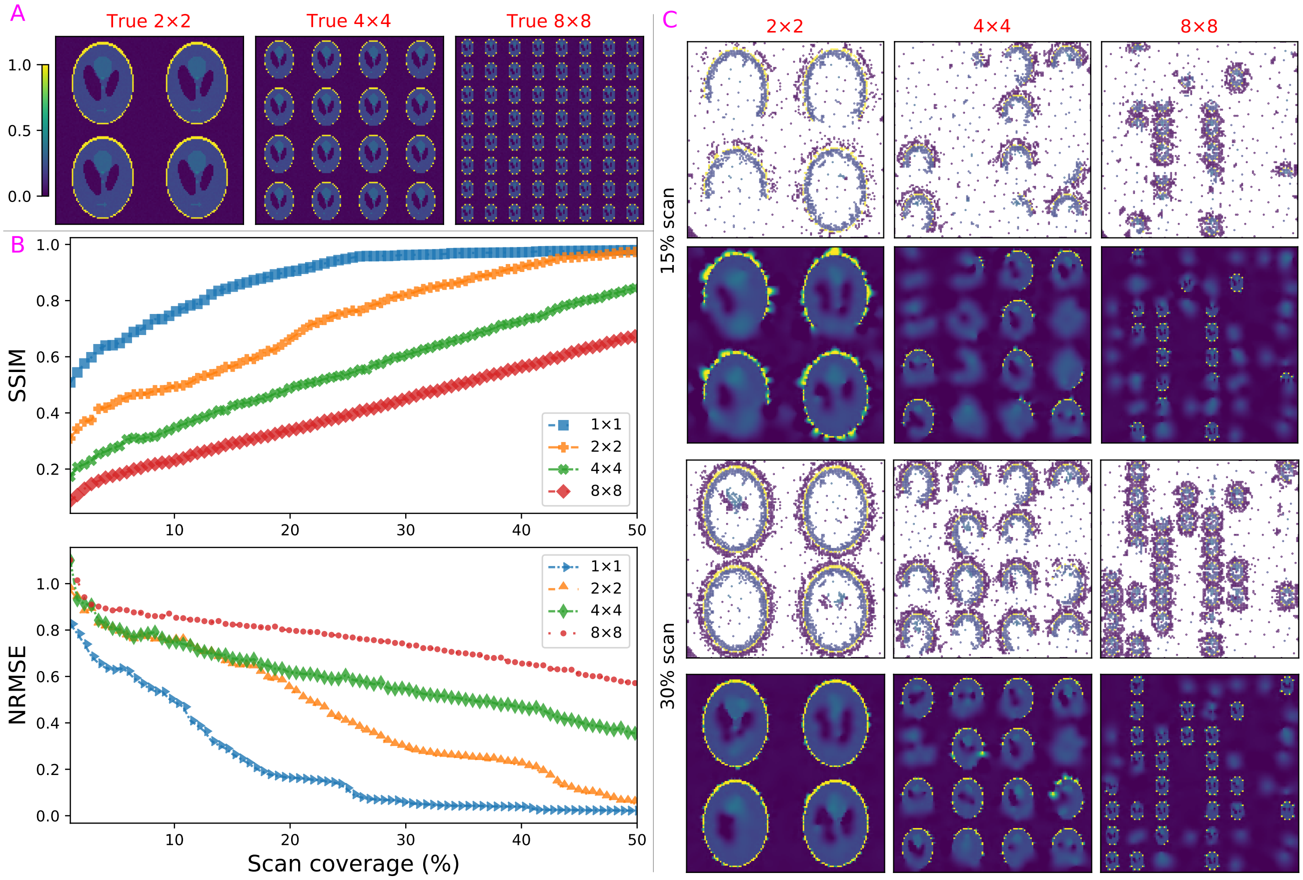

Figure S.7: FAST results for numerical simulations with varying feature densities. (A) shows the full ground truth images for the different experiments. (B) shows the progression in the SSIM and NRMSE f the FAST experiments. (C) shows the scan points and reconstructions for 15 % scan coverage (top two rows) and 30 % scan coverage (bottom two rows). Source data are provided as a Source Data file.

We see that the time required for the FAST scan increases with the increase in the contour of the features. For example, a 16 % scan is sufficient to resolve the outer boundaries in the single Shepp-Logan phantom setting (Figure S.6). In the 2×2 phantom setting, however, we see that outer boundaries are clearly resolved only at around the 30 % mark, which is as expected from the $\approx 2\times$ increase in contour in between these settings.

On the one hand, these results show that FAST is most effective when the scan area consists of isolated features. On the other hand, even for the 8×8, with its dense distribution of features, FAST clearly prioritizes scanning and

resolving the regions of interest over the empty regions, such that the data collected is meaningful. If we further increase the number density of the features, then we get closer to a checkerboard pattern, in which case the FAST method would not present an advantage over a simple raster grid scan.

S.3.3 Effect of feature heterogeneity

In all the experiments with the Shepp-Logan phantom (Section S.3.1 and Section S.3.2), FAST first resolves the higher contrast regions at the boundaries of the phantom, then resolves the lower-contrast eye regions. The lowest-contrast regions, around the mouth and forehead of the phantom, are not resolved clearly until much later in the experiment. On the other hand, these experiments, along with the results in Sections 2.2 and 2.3, show that features with different sizes but similar contrast levels are all prioritized similarly, with no preference based on the feature size. These results suggest that FAST is most useful in settings where all the features, irrespective of their sizes, are at similar levels of contrast. It is possible to address this by preprocessing the intensity input to FAST, such as to increase the contrast levels for specific intensity range of interest, but this requires significant prior information about the experiment. We hope to address this limitation through future work research in the SLADS-NET architecture and feature design.

S.4 Diffraction data analysis for dark-field microscopy

Figure S.8 shows the CoMx and CoMy (as discussed in Section 2.3) calculated from the FAST scan with 20 % coverage in the top two rows. The points marked with $\odot$ and $+$ are located at the top and bottom edges of the bubble, and therefore show additional anomalous diffraction spots. The $\times$ point is in a region without a bubble and has the diffraction pattern at the Bragg angle. The dashed square boxes in the diffraction pattern figures indicate the region of interest (ROI) used for the dark-field image reconstructions (shown in Figure 4 in the main manuscript). The CoM calculations use the regions outside the dashed square boxes as the RoI.as

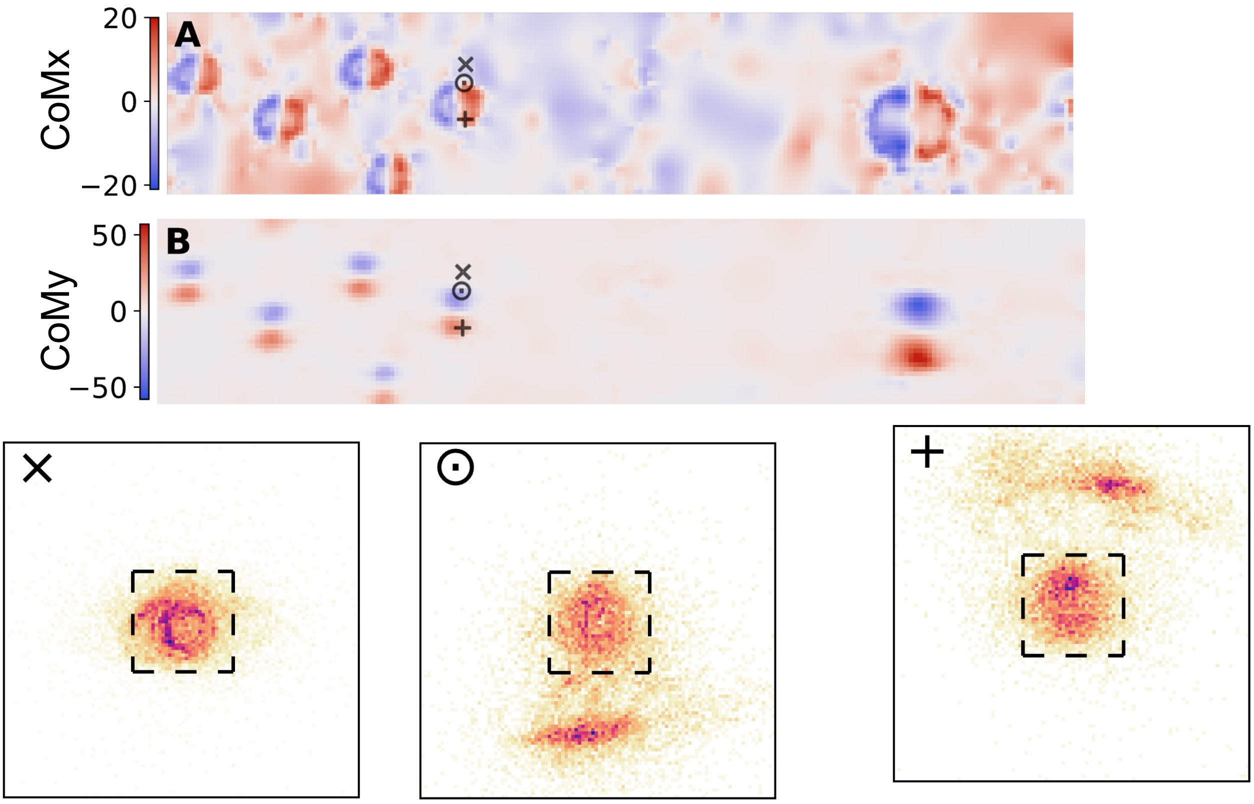

Figure S.8: Example of the analysis performed to quantitatively interpret the surface deformations in the thin film by using the collected diffraction patterns. Suplots (A) and (B) in the top panel (reproduced from Figure 5 in the main manuscript) show respectively the x and y center of mass (CoM) of the region of interest in the diffraction patterns. The diffraction patterns for the points marked with the $\times$, $\odot$, and $+$ are shown in the bottom panel.

S.5 Evolution of the expected reduction in distortion (ERD) during the FAST experiment

Figure S.9 shows the evolution in the ERD during the experimental demonstration discussed in Section 2.3. The ERD initially decreases rapidly, during which point the each batch of 50 points significantly improves the sample reconstruction.The per-iteration change in the ERD is much smaller after 20% coverage.

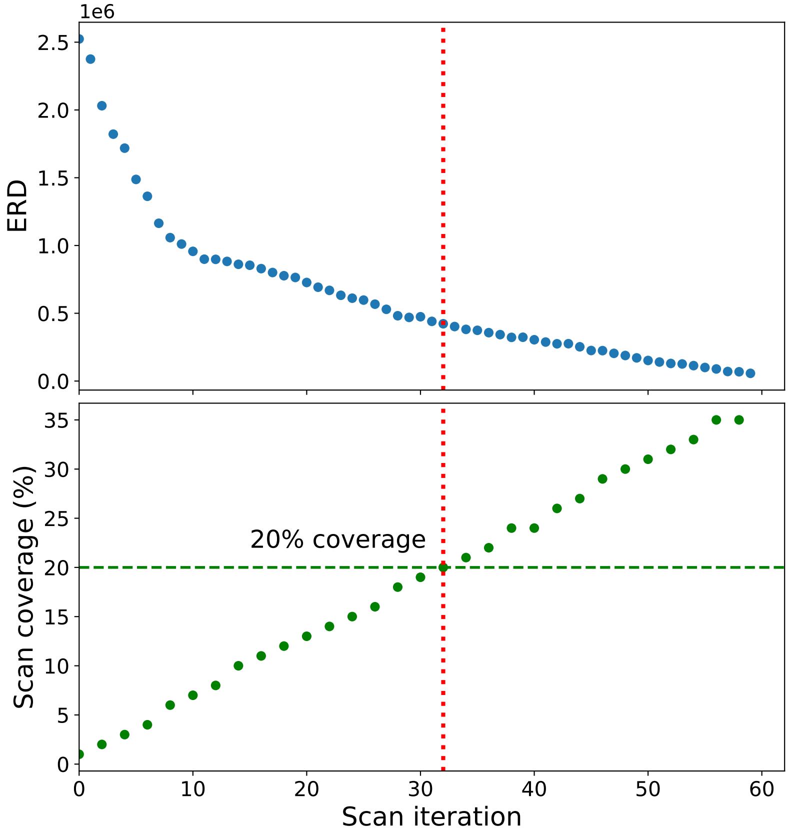

Figure S.9: Evolution in the ERD (top) and the scan coverage (bottom) with the scan iterations in the experimental demonstration. We attain 20 % scan coverage after 32 scan iterations. Source data are provided as a Source Data file.

S.6 Effect of measurement noise on FAST performance

To investigate the effect of measurement noise, we simulated dark-field microscopy experiments at various levels of signal-to-noise ratio (SNR) using the diffraction patterns collected experimentally during the raster scan experiment (discussed as a comparison point in Section 2.3), which we refer to hereon as the "original" dataset. We scaled this original dataset by factors of 10^{-1}, 10^{-2}, and 10^{-3}, then added Poisson noise to these scaled patterns to obtain new intensity data with SNRs of 2, 0.62, and 0.17 respectively. Here, we define the SNR as follows:

$$\mathrm{SNR}(s) = \frac{\|sI_{orig,j}\|_2}{\|sI_{orig,j} - \mathrm{Poisson}\,[sI_{orig,j}]\|_2} \tag{S.1}$$

where $I_{orig,j}$ indicates the original diffraction pattern at position j, s is the scaling factor, $\|.\|_2$ indicates the $L2$ norm, and $I_{new,j} = \mathrm{Poisson}[sI_{orig,j}]$ indices a new rescaled diffraction pattern with added Poisson noise. Example intensity patterns at the different SNR levels are shown in Figure S.10.

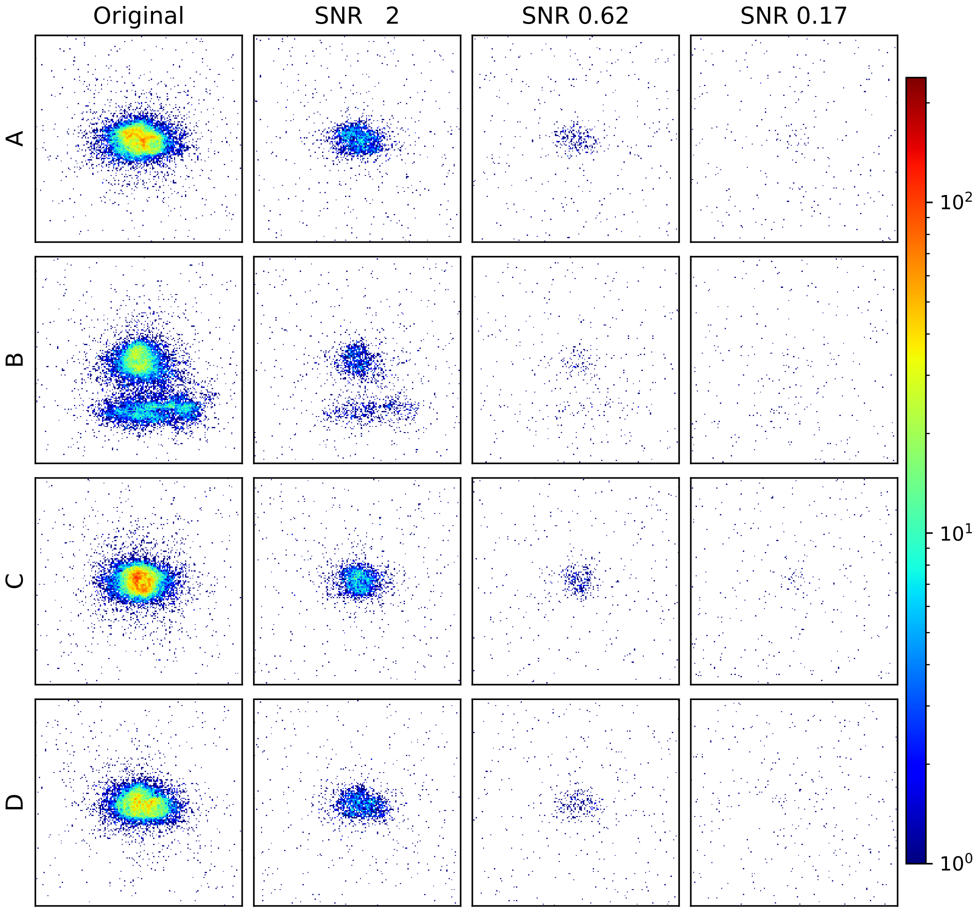

Figure S.10: Examples of the simulated diffraction patterns at different signal-to-noise ratio (SNR) levels. Each row (A,B,C,D) in the leftmost column shows a different diffraction pattern selected from the original experiment dataset, and the other columns show the corresponding diffraction patterns at different SNR levels.

We integrate the diffraction datasets using the usual procedure (discussed in the Section 4.3) to obtain the ground truth microscopy images and during the FAST numerical experiments. Figure S.11 shows the ground truth microscopy images and the FAST measurements and reconstruction at 5 %, 15 %, and 20 % scan coverages for the various SNR levels. From the figure, we can see that FAST shows excellent performance even for low SNR up to the case with the SNR of 0.17. In the latter case, a 20 % FAST scan is not sufficient resolve the features (the

bubbles). We note that sparse measurement methods are generally ineffective for measurements of data with such high levels of point-to-point fluctuations.

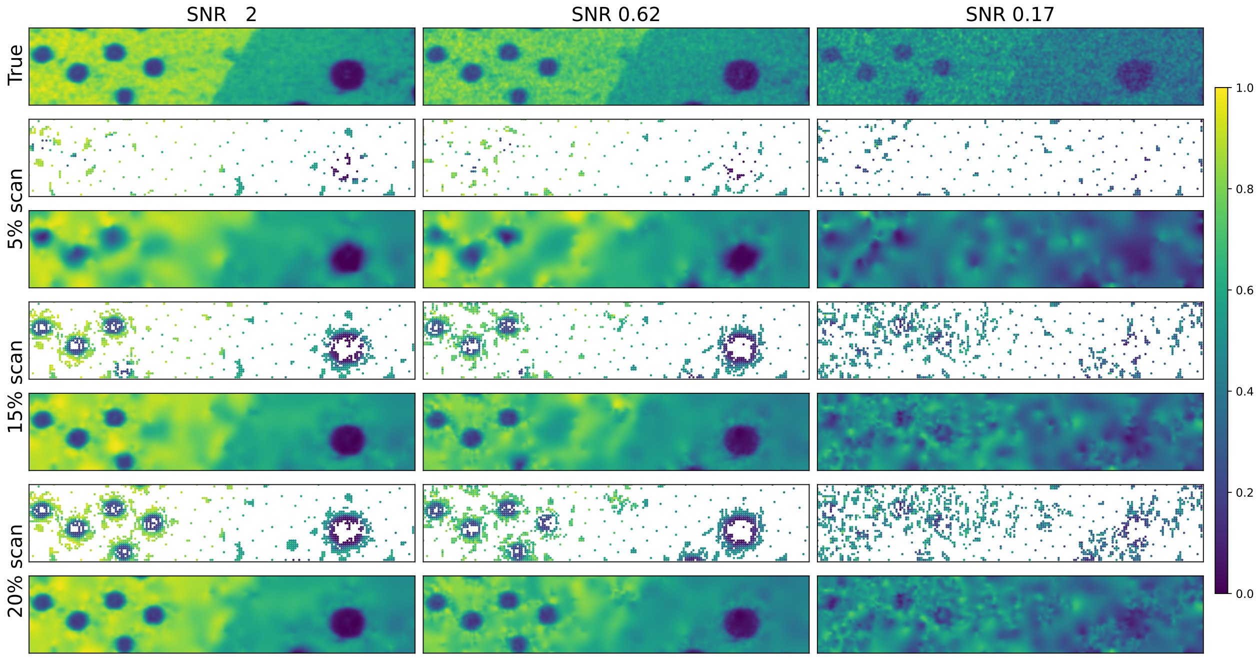

Figure S.11: FAST reconstructions at 5 %, 15 % and 20 % scan coverages for SNR levels of 2, 0.62, and 0.17.

Supplementary References

[1] USC-SIPI image database. [Online; accessed 28-March-2022].

[2] Data files used by the scikit-image project. [Online; accessed 28-March-2022].

[3] Cameraman. https://dome.mit.edu/handle/1721.3/195767 (1978). [Online; accessed 1-Jun-2022].

[4] Kandel, S., Zhou, T. & Cherukara, M. J. Demonstration of an AI-driven workflow for autonomous high-resolution scanning microscopy. fast_smart_scanning : Fast Autonomous Scanning Toolkit, DOI: 10.5281/zenodo.7942774 (2023).
